# Social deprivation and the use of healthcare services over one year by children less than 18 years of age in 2018: A French nationwide observational study

**DOI:** 10.1371/journal.pone.0285467

**Published:** 2023-05-24

**Authors:** Jeanne Pergeline, Sébastien Rivière, Sylvie Rey, Jeanne Fresson, Antoine Rachas, Philippe Tuppin

**Affiliations:** 1 Caisse Nationale de l’assurance Maladie (Cnam), Direction de la Stratégie des études et des Statistiques, Paris, France; 2 Direction de la Recherche, des études, de l’évaluation et des Statistiques (Drees), Paris, France; Aix-Marseille Universite, FRANCE

## Abstract

This study aimed to describe the health status of children and how social deprivation affects their use of healthcare services and mortality. Children living in mainland France were selected from the national health data system (SNDS) on their date of birth or birthday in 2018 (< 18 years) and followed for one year. Information included data on healthcare reimbursements, long-term chronic diseases (LTDs) eligible for 100% reimbursement, geographic deprivation index (FDep) by quintile (Q5 most disadvantaged), and individual complementary universal insurance (CMUc) status, granted to households with an annual income below the French poverty level. The number of children who had at least one annual visit or hospital admission was compared using the ratio of geographic deprivation (rQ5/Q1) and CMUc (rCMUc/Not) after gender and age-standardization. Over 13 million children were included; 17.5% had CMUc, with an increase across quintiles (rQ5/Q1 = 3.5) and 4.0% a LTD (rQ5/Q1 = 1.44). The 10 most frequent LTDs (6 psychiatric) were more common as the deprivation increased. Visits to general practitioners (GPs) were similar (≈84%) for each FDep quintile and the density of GPs similar. The density decreased with increasing deprivation for specialists and visits: paediatricians (rQ5/Q1 = 0.46) and psychiatrists (rQ5/Q1 = 0.26). Dentist visits also decreased (rQ5/Q1 = 0.86) and deprived children were more often hospitalised for dental caries (rQ5/Q1 = 2.17, 2.1% vs 0.7%). Emergency department (ED) visits increased with deprivation (rCMUc/Not = 1.35, 30% vs 22%) but 50% of CMUc children lived in a municipality with an ED vs. 25% without. Approximately 9% of children were admitted for a short stay and 4.5% for a stay > 1 night (rQ5/Q1 = 1.44). Psychiatric hospitalization was more frequent for children with CMUc (rCMUc/Not = 3.5, 0.7% vs 0.2%). Higher mortality was observed for deprived children < 18 years (rQ5/Q1 = 1.59). Our results show a lower use of pediatricians, other specialists, and dentists among deprived children that may be due, in part, to an insufficient supply of care in their area of residence. These results have been used to recommend optimization and specifically adapted individual or area-wide policies on the use of healthcare services, their density, and activities.

## Introduction

Various international studies have shown that poor socio-economic conditions of children, including during early childhood, are associated with poorer health, a higher frequency of chronic diseases, higher mortality, and variations in the use of various healthcare services. Socio-economic conditions are generally estimated using the level of parental income, the geographic deprivation index of the neighbourhood, the lack of insurance coverage or the need for private insurance, and racial and ethnic disparities [1–11]. In a study that included 11 European countries, the likelihood of contacting health services was found to be a function of health status, socioeconomic factors, and healthcare system characteristics [12]. The intensity of use among those having made contact is associated with their health status and sociodemographic characteristics but not the characteristics of the healthcare system. Thus, tackling inequalities at the beginning of life and improving family conditions may optimise child health and development, particularly in the first 1,000 days of life [1, 2, 9, 10]. Despite international policies that aim to address inequalities in the access of children to healthcare services, the gap between the health of children from wealthy and disadvantaged backgrounds continues to grow [13, 14]. However, the proxies and their values for defining economic levels (mainly household income and geographic indices) show varying cut-offs in countries that also have specific healthcare coverage (universal or not). Moreover, study populations have rarely consisted of all or most of a country.

Concerning these aspects, apart from specific diseases or characteristics, there is a lack of regular nationwide or large data to follow specific aspects of child health and healthcare use from 0 to 17 years of age. For example, in France, a national perinatal survey on population samples is carried out at regular intervals [15]. A series of periodic cross-sectional national surveys on child health has also been carried out for various samples of school years and periods [16]. Finally, a national cohort (Elfe) follows 18,000 children selected at birth in 2011 to study determinants of the development, health, and socialization of children from birth to adulthood through a multidisciplinary approach [17]. Thus, faced with a lack of such information, the French Court of Auditors noted that the analysis of a nationwide health and healthcare dataset of children that includes, among others, chronic disease diagnoses and related care, and details the use of healthcare services and the mortality of live-born children according to socioeconomic status is still needed to better understand inequalities in the access of children to healthcare [18].

This could be accomplished using the growing French national health data system (SNDS), which includes the population with public universal health insurance coverage, providing information on healthcare reimbursements for in-hospital medical care or office consultations [19]. It includes a geographical social deprivation index (FDep) based on sociodemographic factors [20] and the status of universal complementary health insurance (CMUc) delivered to people or families with an annual income less than the poverty threshold. Specific studies have shown that children and adults < 60 years of age with CMUc have higher mortality and hospital admission rates than those without [21, 22]. A study of 672,000 women who delivered in France in 2015 reported that 17% had CMUc (< 18 years: 75%, 18–24 years: 35%) [23].

Thus, using the two deprivation markers available in the SNDS, we designed and performed, for the first time, a preliminary national observational study to describe how income and geographical social deprivation are linked to the health status of children < 18 years of age in mainland France, as well as their mortality and use of healthcare services, to develop adequate and targeted policies.

## Methods

### Setting

In many countries, private and public healthcare systems are totally separate. In France, which has universal healthcare coverage, they are “combined”. Health insurance is managed by the national social security system. On average, the social security system reimburses 70% of doctor’s bills and from 15 to 100% of the costs of medication. In addition to this general system, complementary or top-up insurance can be purchased to complete the reimbursement up to the full coverage of all costs. Complementary health insurance plans are a top-up service that cover the remaining costs once social security has allocated its own reimbursement (e.g., the remaining 30% of doctor’s bills). There are a large number of insurance plans available and options must be compared before choosing. Moreover, there is also the universal complementary health insurance (CMUc) plan.

In addition, 100% of the cost of certain consultations is covered by national health insurance for recommended healthcare visits for children. During the first year of life, 11 general practitioner (GP) or paediatrician examinations are recommended. Between the ages of one and two years, two consultations are covered. Thereafter, an examination every two years is planned until the age of 16 years. In France, maternity and child welfare centres (PMI: Protection maternelle et infantile) are public and carry out medical and social prevention for mothers and their children aged < 6 years. A specific French health insurance programme (M’T dents, 2007) covers the costs of dental visits for children aged 6, 9, 12, and 15 years. At these visits, for which an invitation is sent, advice is provided concerning hygiene and oral health and the programme also covers subsequent visits if care is deemed necessary. This programme was expanded to cover a visit for three-year-olds in 2019.

A long-term chronic diseases (LTDs) status guarantees 100% reimbursement for all healthcare expenditures related to the LTD for at least five years upon request by the GP of the patient; it can be requested or renewed according to disease evolution and clinical status. LTDs include many chronic diseases that require costly, regular, and long-term care and are potentially life-threatening or could lead to disabilities. The list of LTDs is periodically published by decree based on expertise from the HAS (Haute Autorité de Santé, French National Authority for Health) [24, 25]. Diagnoses are filed and validated after medical exams and, eventually, hospitalisation, based on International Classification of Diseases 10^th^ revision (ICD-10) codes using national recommendations for diagnosis, treatment, and follow-up for such diseases and a guide is sent to the patient [25].

### Design and study population

This retrospective observational study included children < 18 years of age in 2018 and followed up for one year between 2018 and 2019 according to their births or birthdays. In mainland France (population of 64.9 million), there were 13.99 million children < 18 years of age on the first of January 2019, according to the INSEE [Institut national de la statistique et des études économiques, National Institute for Statistics and Economic Studies] [26]. Those excluded from the analysis included children who received no refund during the one year follow-up period and those of the same sex resulting from a multiple birth (twins, triplets, etc.) who could not be unambiguously identified in the database, as those with a false number of linkages or those lacking critical information, such as the month of birth (N = 13.5 million remaining). For the sake of homogeneity, we also excluded children of different sexes resulting from multiple births (N = 13.219 million remaining). We also excluded children whose month of birth could not be identified (N = 13.214 million remaining) and those who died in 2018 before the beginning of the follow-up (N = 13,213 million remaining). Finally, those who died during the one-year follow-up study period were analysed only for the mortality study and excluded from the others. Thus, 13.211 million children were included (94.4%). Overall, this study concerned singleton children living in mainland France with national health insurance coverage who had at least one healthcare reimbursement from health insurance in 2018.

### Data source

The SNDS collects individual information from the various French health insurance schemes [19]. It does not record office consultation diagnoses or the results of clinical examinations and investigations. Nevertheless, it includes information on the presence of LTDs. Using a pseudonymised identification number, all this information is linked, via the national hospital discharge database, to data concerning public and private hospital stays: short-stay hospitalisations (SSHs), stays in psychiatric hospitals or rehabilitation facilities, and home hospitalisation. Normal stays in hospitals/maternity units for childbirth without the need for neonatal care were excluded. Information is also collected for various SSH units, such as the intensive care unit (ICU, paediatric or adult), neonatology, and emergency department (ED) visits followed or not by admission for SSHs. The primary hospital diagnoses for SSHs (including day hospitalisation) and LTD diagnoses are coded according to the International Classification of Diseases 10^th^ revision (ICD-10). Data concerning the presence of an ED in the municipality were current as of December 31, 2019, and available from the DREES (Direction de la recherche, des études, de l’évaluation et des statistiques of the French Health Ministry).

### Outcome measures

The primary outcome was the prevalence of at least one LTD with details concerning the disease chapter in the ICD-10 and the most frequent diagnosis. The second outcome was the frequency of outpatient visits, defined as reimbursement for a service provided by a primary healthcare professional outside the hospital, hospital outpatient consultation, or visits to an ED, hospital stays, and mortality. The primary diagnosis for each SSH was also analysed to identify the frequency of acute and chronic diagnoses. Primary diagnoses with an ICD-10 code ranging from Z00 to Z99 “Factors influencing health status and contact with healthcare services” concerns individuals using healthcare services for examinations. We used the related diagnosis of the stay, if available, to obtain information about the disease. Among SSHs, 313,098 within the Z group had a primary diagnosis and for 83,552, the related diagnosis was not indicated (27%).

### Social deprivation

The FDep is divided into quintiles computed in 2015 (Q1: least deprived, Q5: most deprived) [20]. It was constructed at the “commune” scale (smallest administrative unit, 36,000 units in mainland France and similar to a municipality) according to four factors resulting from data published by the INSEE: average household income, the percentage of high school graduates (graduates in France who passed the Baccalaureate exam [national exam required to obtain a high school diploma]) among inhabitants aged 15 and over, the percentage of blue-collar workers in the active population, and unemployment levels. In addition, we calculated the primary healthcare practitioner density between quintiles (per 100,000 inhabitants). Among the study population, the FDep was missing for 1.1% of the children, mainly due to the absence of information on the commune of residence.

The CMUc is a renewable benefit granted for one year to people who have had a stable and regular residence in France for at least three months. The household can include the applicant, his/her spouse/partner, and children. In 2018, the annual income limit was 8,810 € per annum for a single person, with an increase according to the number of people in the household. This limit is below the French poverty threshold, defined as 50% of the median income, which was 10,620 € in 2018. The CMUc enables beneficiaries to access treatment without advancing costs (Practitioners are paid directly by the health insurance scheme) with a reimbursement of 100% and without extra fees. In 2018, CMUc beneficiaries accounted for 8.2% of the resident population in France [27], were more likely to live in a single-parent household (33% vs 10.4%), in a household in which the reference person was a laborer (40% vs 30%), an employee (30% vs 15%), or unemployed (41% vs 5%) and who had an educational level below upper secondary school for 79% vs 52% in the general population. Children were classified as being a CMUc beneficiary if they had at least one specific outpatient reimbursement covered by the CMUc in 2018 or 2019. The CMUc coverage status could not be identified for children who did not have any outpatient reimbursements and was classified as a missing value (0.1% of our population). Nevertheless, most infants who did not have any reimbursements mainly died in the hospital after birth, without being discharged alive from the hospital, and consequently had no outpatient reimbursement with CMUc information.

### Analyses

We described the children, the characteristics of the communities in which they lived, with their healthcare density and utilization, and mortality according to the two social deprivation markers selected. The rates of children with a LTD was described globally (at least one LTD) and for the 10 most frequent diagnoses, as well as overall mortality. For each deprivation group, the rates of visits to healthcare services during the year were calculated following birth (for those born in 2018) and between birthday months for the others (due to anonymization). Then, we calculated the overall risk ratios for those with CMUc and those without (rCMUc/No) and the most and least deprived quintiles of the FDep (rQ5/Q1), with gender and age-standardization. For calculation of the time between an ED visit and consultation (0 to 30 days, S1 Table) with a general practitioner (GP) or paediatrician, one month before and after the one-year follow-up were included. The global one-year live birth mortality rate (0–1 year) and the population were also reported for the deprivation levels.

Data on the SSH primary diagnoses are expressed as the rate among the population that had at least one SSH stay after gender and age-standardization using the same population. To assess the intensity of healthcare use, the median and interquartile range (IQR) of the number of utilizations are reported for children who used healthcare services at least once during the year.

SAS software (version 7.13, SAS Institute Inc, Cary, NC, USA) and R software (3.4.3) were used for statistical analysis.

### Ethics approval

A specific ethics committee approval was not required for this study. The French national health insurance (CNAM) in charge of the SNDS (système national des données de santé) has permanent access to the pseudonymized reimbursement data in application of the provisions of articles R. 1461‐12 et seq. of the French Public Health Code, with rules and criteria similar to those of the Helsinki declaration. The CNAM has permanent full access to the SNDS by decree (Décret n° 2016–1871 du 26 décembre 2016 relatif au traitement de données à caractère personnel dénommé « système national des données de santé ») The CNAM has authorization to perform studies based on SNDS data from the CNIL (National independent Commission for Computing and Freedom, the French data protection agency as sensitive information). All methods were carried out in accordance with relevant guidelines and regulations. Detailed information on the process and the system may be found in reference 19.

## Results

### Population and area characteristics

Among the 13,211 million children included (51.2% boys), the social deprivation index (FDep) analysis concerned 13.06 million and that of the CMUc status 13.20 million (Table 1). Age and gender distributions were similar between quintiles of the FDep. CMUc was identified for 17.5% of children with a median age of eight years (IQR 4–12) and for those without CMUc, nine years (IQR 4–13). As expected, the CMUc frequency increased with the deprivation index quintile (Q5:29.7%, rQ5/Q1 = 3.49). The proportion of children living in a municipality with an ED was 29.5% but differed mainly for those with CMUc (50%) versus those without (25%). The density of healthcare professionals varied between quintiles of the FDep: it increased across quintiles for nurses (rQ5/Q1 = 1.59), was stable for GPs (rQ5/Q1 = 1.00), and decreased for other healthcare professionals, mainly psychiatrists (rQ5/Q1 = 0.26) and pediatricians (rQ5/Q1 = 0.40).

**Table 1:** Characteristics and most frequent long-term diseases of children < 18 years of age in 2018 and followed for one year after their birthday or birth by geographical social deprivation index and complementary universal health insurance coverage and ratios standardized by gender or age.

		Social deprivation index (quintiles) (least deprived Q1, most deprived Q5)					CMUc		Ratios	
	Total	Q1	Q2	Q3	Q4	Q5	Without	With	Q5/Q1	CMUc/No CMUc
N (millions)	13.211	2.578	2.646	2.595	2.580	2.661	10.880	2.319		
% line (missing exluded)	100	19.5	20.0	19.6	19.5	20.1	82.4	17.5		
	%	%	%	%	%	%	%	%		
**Boys**	51.2	51.2	51.1	51.2	51.2	51.1	51.1	51.4	1.00	1.01
**Age (year)**										
Median [IQR]	9 [4–13]	9 [4–13]	9 [4–13]	9 [4–13]	9 [4–13]	9 [4–13]	9 [4–13]	8 [4–12]		
< 1	5.1	5.3	5.1	5.0	4.9	5.1	5.0	5.6	0.97	1.12
1- <2	5.3	5.4	5.3	5.2	5.1	5.3	5.1	6.3	0.99	1.23
2 - <5	16.4	16.4	16.5	16.3	16.1	16.6	15.8	19.1	1.01	1.21
5 - <10	28.4	28.3	28.5	28.4	28.4	28.5	28.2	29.7	1.01	1.05
10 - <14	22.7	22.6	22.6	22.7	22.9	22.6	23.1	20.7	1.00	0.89
14 - <18	22.1	22.0	22.0	22.3	22.5	22.0	22.9	18.7	1.00	0.82
**CMUc**	17.5	8.5	13.0	17.4	19.8	29.7	-	-	3.49	
**Living in a municipality with ED**	29.5	21.5	24.8	34.0	32.2	35.9	25.1	50.1	1.67	1.99
Missing	1.0	0.1	0.1	0.6	0.5	0.5	1.2	0.3		
**Professional density** p 100 000										
GP	73.6	72.4	75.4	75.6	72.1	72.4			1.00	
Pediatrician	2.8	4.2	2.9	3.2	2.0	1.7			0.40	
Ophthalmologist	4.9	5.3	4.6	5.9	4.6	4.4			0.84	
Psychiatrist	6.2	11.3	6.2	6.7	3.8	2.9			0.26	
ENT	2.0	2.1	1.8	2.5	1.9	1.5			0.74	
Endocrinologist	0.8	1.0	0.8	0.9	0.6	0.6			0.58	
Pulmonologist	1.2	1.2	1.2	1.5	1.4	1.0			0.88	
Gastroenterologist	2.0	2.2	1.9	2.5	1.8	1.8			0.81	
Dentist	54.5	67.2	53.8	55.0	49.1	47.5			0.71	
Nurse	147.5	106.3	138.4	159.2	163.8	168.8			1.59	
Physiotherapist	108.9	132.3	118.2	114.1	97.5	83.1			0.63	
**At least one LTD** *	4.0	3.2	3.7	4.1	4.3	4.6	3.6	5.8	1.44	1.61
In boys **	4.6	3.6	4.4	4.8	5.0	5.4	4.2	6.9	1.50	1.64
In girls **	3.3	2.7	3.1	3.4	3.5	3.7	3.0	4.7	1.37	1.57
**Most frequent LTD (ICD-10)** *										
Pervasive developmental disorders (F84)	0.53	0.44	0.52	0.55	0.55	0.59	0.45	0.92	1.34	2.04
Asthma (J45)	0.24	0.23	0.21	0.23	0.21	0.30	0.22	0.31	1.30	1.41
Specific developmental disorders of speech and language (F80)	0.17	0.11	0.14	0.17	0.22	0.23	0.15	0.28	2.09	1.87
Epilepsy (G40)	0.17	0.14	0.15	0.17	0.18	0.20	0.15	0.24	1.43	1.60
Unspecified mental retardation (F79)	0.16	0.11	0.14	0.18	0.17	0.20	0.13	0.30	1.82	2.31
Type 1 diabetes mellitus (E10)	0.15	0.13	0.15	0.15	0.15	0.17	0.14	0.18	1.31	1.29
Scoliosis (M41)	0.15	0.15	0.16	0.15	0.15	0.14	0.16	0.10	0.93	0.63
Specific developmental disorders of scholastic skills (F81)	0.12	0.07	0.11	0.12	0.15	0.14	0.10	0.19	2.00	1.90
Mixed disorders of conduct and emotions (F92)	0.10	0.05	0.09	0.11	0.12	0.12	0.08	0.22	2.40	2.75
Mixed specific developmental disorders (F83)	0.09	0.04	0.08	0.11	0.12	0.09	0.07	0.18	2.25	2.57
**Mortality by age p1000**										
< 1	2.06	1.61	1.70	2.02	2.08	2.96	0.73	1.08	1,84	1.48
1- <2	0.24	0.16	0.20	0.32	0.20	0.35	0.19	0.42	2,19	2.21
2 - <5	0.11	0.08	0.11	0.12	0.12	0.13	0.10	0.15	1,63	1.50
5 - <10	0.06	0.06	0.06	0.05	0.08	0.07	0.06	0.10	1,17	1.67
10 - <14	0.07	0.05	0.09	0.05	0.08	0.08	0.06	0.09	1,60	1.50
14 - <18	0.15	0.15	0.15	0.14	0.14	0.18	0.13	0.25	1,20	1.92
**Total** **	0.21	0.17	0.19	0.21	0.20	0.27	0.12	0.21	1,59	1.75

*Note*.

*Standardized by age and gender

** Standardized by age, LTD: long term disease, CMUc: complementary universal health insurance coverage, ICD-10: international classification of diseases

### Chronic diseases

At least one LTD was found for 4.0% of all children (4.6% boys, 3.3% girls) and the prevalence increased with social deprivation (rQ5/Q1 = 1.44). Those with an LTD (median age 10 years [IQR 6–14]) were older than those without (8 years [4–13]). Each of the 10 most frequent LTDs (Table 1) became more frequent as the deprivation increased (ratios > 1), except for scoliosis (rQ5/Q1 = 0.93). The highest ratios were found for psychiatric LTDs: pervasive developmental disorders (rQ5/Q1 = 1.34), mixed disorders of conduct and emotions (rQ5/Q1 = 2.40), unspecified mental retardation (rQ5/Q1 = 1.82), and mixed specific developmental disorders (rQ5/Q1 = 2.25).

### Mortality

The overall one-year mortality for children < 18 years was 0.21 p 1000 (2.06 p 1000 for < 1 year followed by a relatively large decrease (10 - < 14 years = 0.07 p 1000, 14 - < 18 years = 0.15). By FDep quintile, overall mortality was higher as the level of deprivation increased (rQ5/Q1 = 1.59), especially between 0–2 years of age (rQ5/Q1 = 2.19). For those with CMUc, overall mortality (rCMUc/No = 1.75) was also higher. It was equal to 2.2 for children from 1 to 2 years of age, but less for those < 1 year (1.28). However, 61% of the children who died before the age of one, mainly in the first month, had received no outpatient refund. This suggests death in the hospital without discharge and, consequently, the CMUc status could not be found.

### Outpatient visits

**Fig 1:**
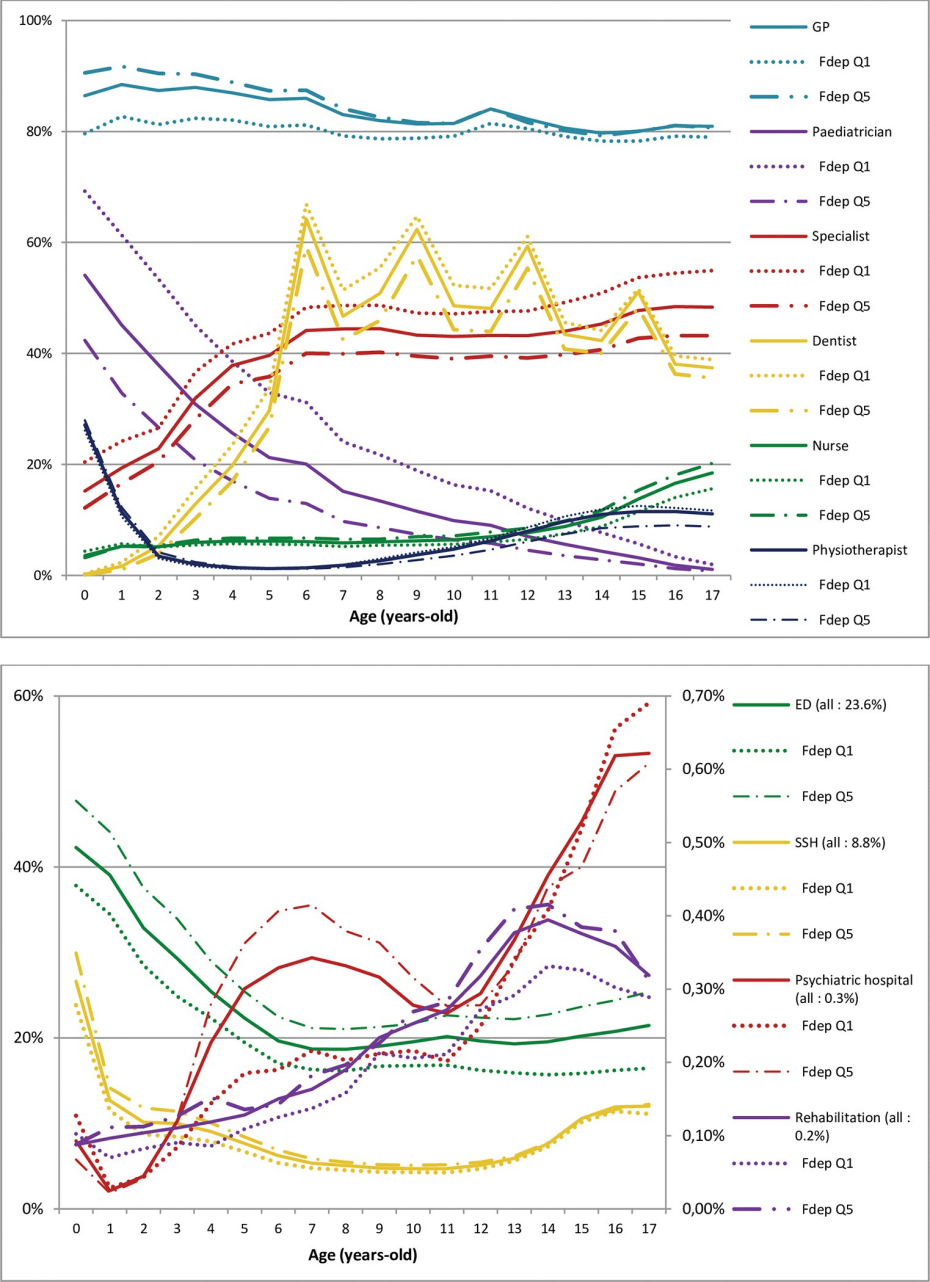
Proportion of children with at least one visit to a physician, emergency department, or hospitalisation among all children in 2018 by age and geographical social deprivation index (least deprived Q1, most deprived Q5). FDep: geographical social deprivation index.

**Fig 2:**
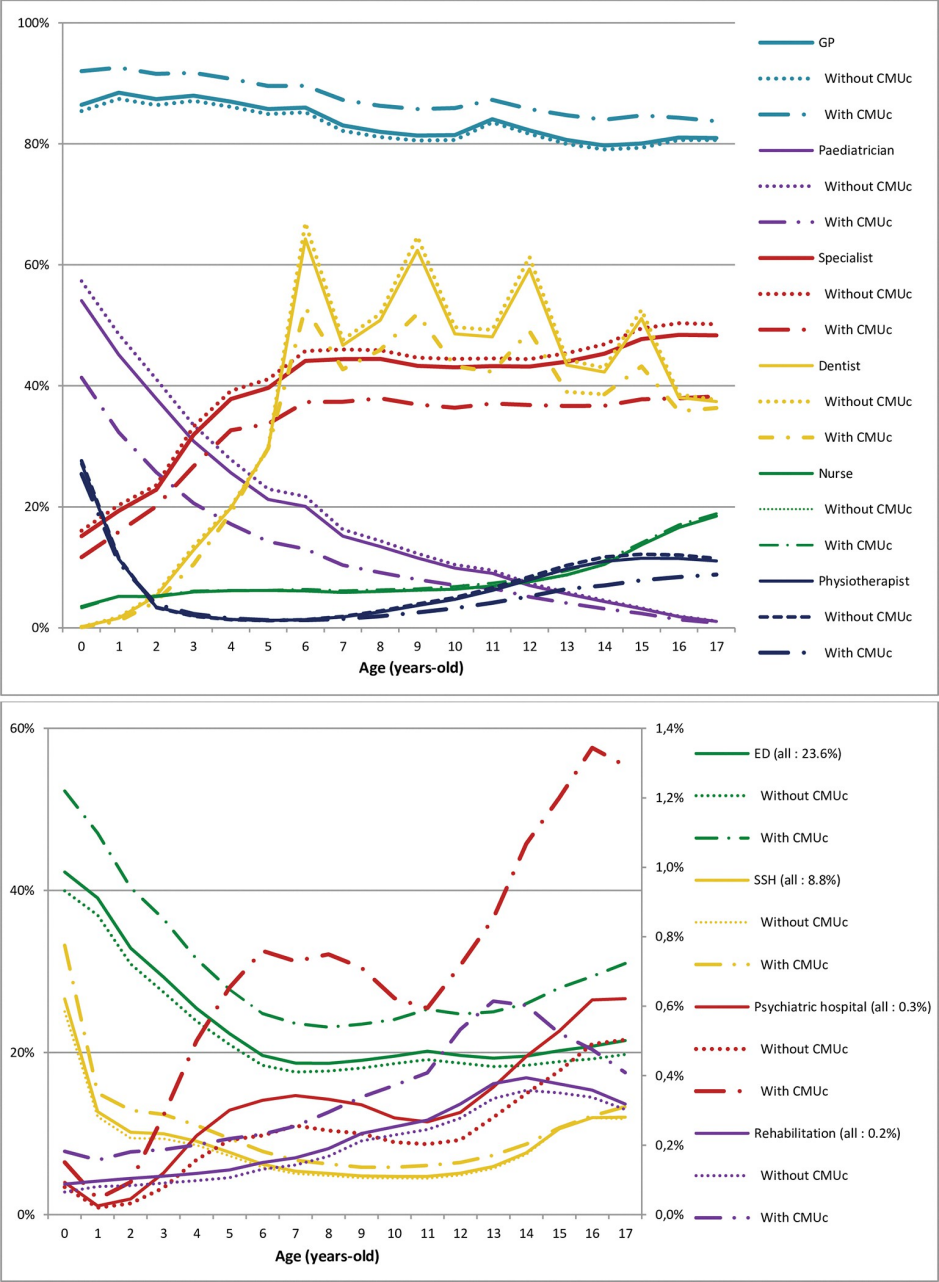
Proportion of children with at least one visit to a physician, emergency department, or hospitalisation among all children in 2018 by age and complementary universal health insurance coverage (CMUc). CMUc: complementary universal health insurance coverage.

The proportion of children who consulted a GP or paediatrician at least once during the year was similar between FDep quintiles (approximately 88%), ratios close to one at each age (Table 2, Figs 1 and 2). For pediatrics alone, the consultation frequency decreased regularly by quintile (Q5 = 11.9%, rQ5/Q1 = 0.46). Thus, children in the Q1 quintile were slightly less likely to visit a GP, but more likely to visit a paediatrician, than those in the Q5 quintile until 10 years of age. Children living in deprived areas were also less likely to have visited other specialists, overall (rQ5/Q1 = 0.80), and for all ages with differences according to specialist. The highest differences were for psychiatrists (rQ5/Q1 = 0.26). For dentists, the frequency of visits was globally the lowest for deprived children (rQ5/Q1 = 0.86). The overall visit rate was approximately 30% at five years of age with peaks at 6, 9, 12, and 15 years of approximately 60% and a plateau of approximately 50% at other ages, with a slight decrease during adolescence. The rate of visits to a nurse was slightly higher for more deprived than less deprived children for all ages (rQ5/Q1 = 1.22). For physiotherapists, the ratio was also low (rQ5/Q1 = 0.85) and the difference was mainly found for adolescents, with no difference among the youngest.

**Table 2:** Use of outpatient and hospital healthcare services by children < 18 years of age and followed for one year after their birth or birthday in 2018 by social deprivation index and complementary universal health insurance coverage and ratios standardized by gender and age.

		Social deprivation index (quintiles) (least deprived Q1, most deprived Q5)					CMUc		Ratios	
	Total	Q1	Q2	Q3	Q4	Q5s	Without	With	Q5/Q1	CMUc/ No CMUc
N (millions)	13.211	2.578	2.646	2.596	2.580	2.662	10.880	2.319		
At least once during the year	%	%	%	%	%	%	%	%		
**Outpatient visit**										
GP or pediatrician*	88.0	87.9	88.9	88.3	87.8	87.2	87.8	89.9	0.99	1.02
Median [IQR]**	3 [2–6]	3 [2–6]	3 [2–6]	3 [2–6]	3 [2–6]	3 [2–6]	3 [2–5]	4 [2–7]		
GP *	83.6	80.1	84.7	84.0	84.6	84.6	82.8	87.6	1.06	1.06
Pediatrician*	17.3	25.6	18.0	17.2	13.7	11.9	18.5	12.1	0.46	0.65
Specialists (except pediatrician)*	39.5	44.2	41.0	39.3	37.4	35.5	40.9	32.9	0.80	0.80
Ophthalmologist*	24.7	26.5	25.7	24.8	24.1	22.6	25.7	20.5	0.85	0.80
Dermatologist*	7.1	9.8	7.7	6.8	5.8	5.2	7.5	4.6	0.53	0.61
ENT*	7.0	8.3	7.3	6.8	6.4	6.3	7.3	6.2	0.76	0.85
Cardiologist*	1.2	1.3	1.3	1.3	1.1	1.1	1.2	1.1	0.85	0.92
Pulmonologist*	1.2	1.0	1.2	1.4	1.2	1.1	1.2	1.1	1.10	0.92
Psychiatrist*	1.0	1.9	1.1	1.0	0.6	0.5	1.1	0.7	0.26	0.64
Dentist*	37.3	39.7	38.6	37.4	36.4	34.3	38.2	33.0	0.86	0.86
Nurse*	8.1	7.2	7.8	8.1	8.5	8.8	8.0	8.4	1.22	1.05
Physiotherapist*	7.1	7.3	7.8	7.3	6.8	6.2	7.3	5.6	0.85	0.77
**Emergency department (ED) visit**										
All ED visit*	23.6	20.0	22.0	24.2	24.8	26.9	22.2	30.0	1.35	1.35
Median [IQR]**	1 [1–2]	1 [1–2]	1 [1–2]	1 [1–2]	1 [1–2]	1 [1–2]	1 [1–2]	1 [1–2]		
ED visit not followed by SSH*	21.9	18.6	20.4	22.5	23.0	25.0	20.6	28.0	1.34	1.36
ED visit followed by SSH > 1 night*	2.9	2.2	2.6	2.9	3.1	3.5	2.6	4.1	1.59	1.58
**Hospital stays**										
Short stay hospital (SSH)*	8.8	7.9	8.6	8.8	9.0	9.5	8.4	10.4	1.20	1.24
Median [IQR]**	1 [1–1]	1 [1–1]	1 [1–1]	1 [1–1]	1 [1–1]	1 [1–1]	1 [1–1]	1 [1–1]		
SSH more than one night *	4.5	3.6	4.2	4.5	4.7	5.2	4.1	6.0	1.44	1.46
Pediatric ICU*	0.09	0.1	0.1	0.1	0.1	0.1	0.1	0.1	1.00	1.00
Neonatology*	0.6	0.5	0.5	0.6	0.6	0.7	0.5	0.7	1.40	1.40
Neonatology with ICU*	0.2	0.2	0.2	0.2	0.2	0.3	0.2	0.3	1.50	1.50
Psychiatric hospital*	0.3	0.3	0.3	0.4	0.3	0.3	0.2	0.7	1.00	3.50
Rehabilition unit*	0.2	0.2	0.2	0.2	0.2	0.2	0.2	0.3	1.00	1.50

*Note*.

CMUc: complementary universal health insurance coverage

SSH: short stay hospitalisation

ED: emergency department

ICU: intensive care unit

* Standardized on gender and age

** Median and IQR were always calculated for children with at least one visit or hospitalisation

### ED visits and hospital stays

The proportion of children who made at least one visit to an ED (23.6%) during the year was higher for children living in the most deprived quintile (26.9%) than for those in the least deprived (20.0%; rQ5/Q1 = 1.35) (Table 2). Differences were consistent across ages (Fig 1). When only ED visits followed by a SSH stay > 1 day were considered, the ratio was higher (1.59), suggesting that deprived children were more often hospitalized following an ED visit. There was also a slightly higher proportion of the most deprived children with at least one SSH stay (Q5 9.5% vs Q1 7.9%, rQ5/Q1 = 1.20), mainly observed before four years of age. This was also true for SSH stays > 1 day (Q5 5.2% vs Q1 3.6%, rQ5/Q1 = 1.44). Admission to a neonatology ICU was more frequent for deprived children (rQ5/Q1 = 1.50). There was no increase in the admission frequency across FDep quintiles for psychiatric hospitals.

**Fig 3:**
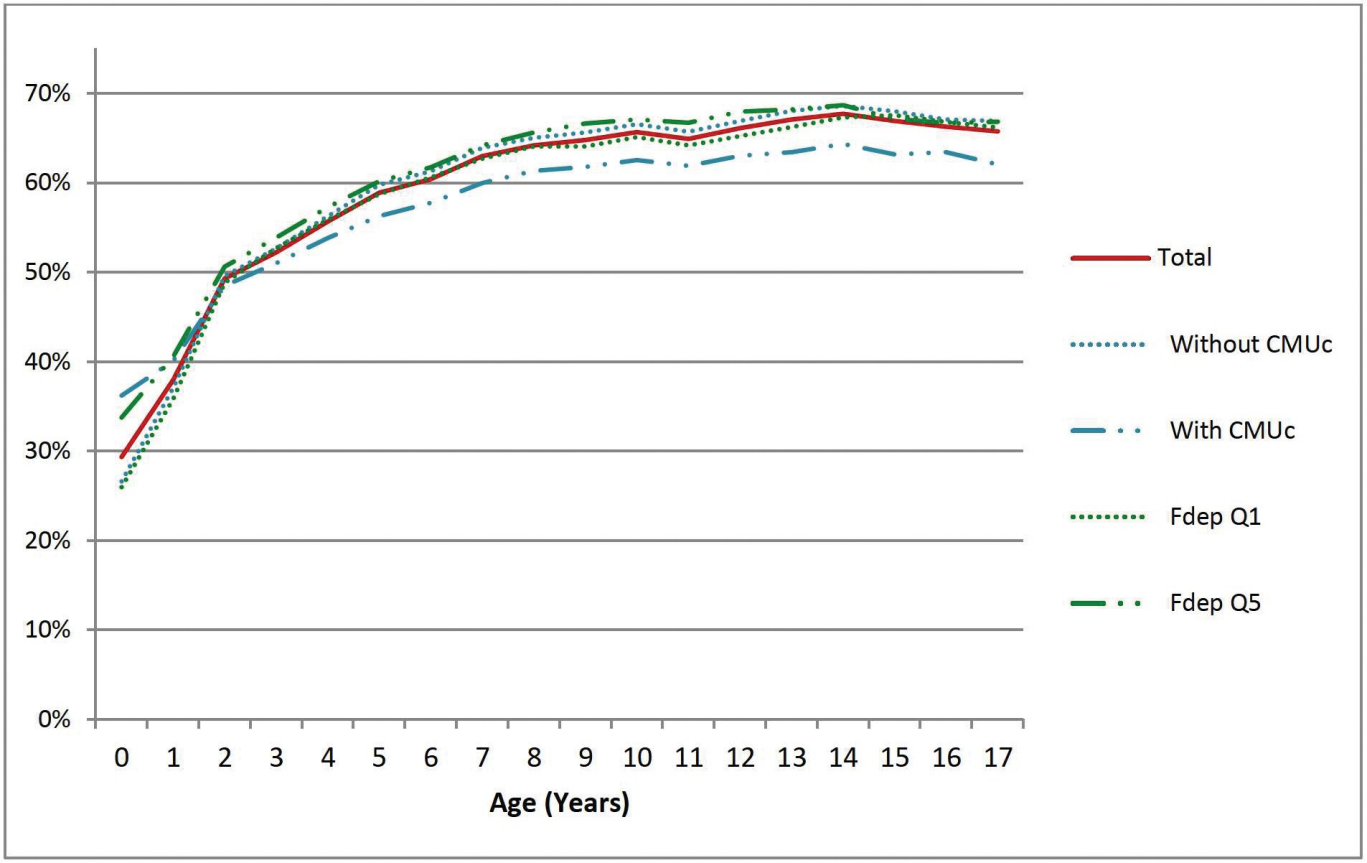
Proportion, by age, of ED visits occurring without a GP or paediatrician visit between 30 days and the day before by complementary universal health insurance coverage (CMUc) and the geographical social deprivation index (FDep). CMUc: complementary universal health insurance coverage, FDep: geographical social deprivation index.

Among all ED visits, the proportion of children who visited a GP or paediatrician on the same day was 8.8% (S1 Table). This proportion was higher when the ED visit was followed by a SSH than not (14.7% *vs* 8.1%), with a slight decrease with increasing deprivation (). Nevertheless, it is not possible to know whether the consultation occurred before or after the ED visit. Globally, 59.7% of children had no consultation between 30 days and the day before the ED visit and 49.7% when there was a SSH admission. This value was similar across quintiles (Fig 3). After an ED visit without admission, 60% of children also had no GP or paediatrician consultations during the following 30 days.

### Hospital diagnosis

The most frequently occurring diseases coded as primary diagnoses for SSH stays were digestive diseases (19.1% of the children with at least one stay) and respiratory diseases (17.0%) (Table 3). Digestive system diseases were less frequent among deprived children (rQ5/Q1 = 0.92), in contrast to respiratory diseases (rQ5/Q1 = 1.07). Pregnancy, childbirth, and puerperium diseases were found for 0.5%, with higher rates for deprived children (rQ5/Q1 = 2.00). A higher rate for deprived children was also found for diagnoses of certain infectious and parasitic diseases (rQ5/Q1 = 1.40), diseases of the circulatory system (rQ5/Q1 = 1.20), and psychological and behavioural disorders (rQ5/Q1 = 1.15 and = 2 at six years). Conversely, a ratio < 1 was found for other diseases, such as neoplasm (rQ5/Q1 = 0.76), diseases of the ear and mastoid process (rQ5/Q1 = 0.85), diseases of the blood and blood-forming organs, disorders involving the immune system (rQ5/Q1 = 0.86), and congenital malformations, deformations, and chromosomal abnormalities (rQ5/Q1 = 0.88).

**Table 3:** Proportion of principal diagnoses by ICD-10 chapter and 20 most frequent principal diagnoses by ICD-10 among hospitalisations of children < 18 years-old and followed for one year after their birthday in 2018 according to social deprivation index and complementary universal health insurance coverage (CMUc) and ratios standardized by gender and age.

	Total	Social deprivation index (quintiles) (least deprived Q1, most deprived Q5)					CMUc		Ratios	
		Q1	Q2	Q3	Q4	Q5	Without	With	Q5/Q1	CMUc/Not CMUc
Children with at least one SSH hospitalisation N (millions)	1.158	0.205	0.229	0.228	0.230	0.252	0.909	0.247		
**ICD 10 chapter**	%	%	%	%	%	%	%	%	%	%
• Diseases of the digestive system	19.1	19.5	19.4	19.3	19.2	18.0	19.7	15.2	0.92	0.77
• Diseases of the respiratory system	17.0	16.5	17.0	16.9	16.9	17.6	16.9	17.7	1.07	1.05
• Injury, poisoning and consequences of external causes	11.2	11.0	11.2	11.3	11.3	11.1	11.0	12.3	1.01	1.12
• Symptoms, signs and abnormal clinical and laboratory findings,	9.2	8.4	8.7	9.1	9.4	10.2	8.7	11.1	1.21	1.28
• Diseases of the genitourinary system	8.9	9.0	8.7	8.5	8.7	9.7	8.5	10.2	1.08	1.20
• Certain conditions originating in the perinatal period	6.4	6.6	6.3	6.4	6.5	6.4	6.4	6.5	0.97	1.02
• Certain infectious and parasitic diseases	6.1	5.0	5.7	6.3	6.5	7.0	5.9	7.0	1.40	1.19
• Factors influencing health status and contact with health services	5.8	5.7	6.0	5.8	5.7	5.7	5.8	5.8	1.00	1.00
• Congenital malformations, deformations, and chromosomal abnormalities	5.4	5.6	5.6	5.6	5.3	4.9	5.6	4.7	0.88	0.84
• Diseases of the ear and mastoid process	4.5	4.7	4.9	4.5	4.6	4.0	4.9	3.4	0.85	0.69
• Diseases of the musculoskeletal system and connective tissue	3.4	3.6	3.5	3.4	3.2	3.2	3.5	3.1	0.89	0.89
• Diseases of the skin and subcutaneous tissue	3.0	2.9	3.0	3.0	2.9	3.2	3.0	3.1	1.10	1.03
• Endocrine, nutritional and metabolic diseases	2.9	3.3	2.8	2.8	2.7	2.9	2.8	3.2	0.88	1.14
• Mental and behavioural disorders	2.7	2.6	2.6	2.6	2.8	3.0	2.4	4.4	1.15	1.83
• Diseases of the nervous system	2.7	2.6	2.7	2.7	2.7	2.8	2.6	3.1	1.08	1.19
• Neoplasms (malignant or benign)	1.5	1.7	1.6	1.5	1.5	1.3	1.6	1.2	0.76	0.75
• Diseases of the eye and adnexa	1.3	1.3	1.3	1.4	1.3	1.2	1.4	1.2	0.92	0.86
• Diseases of the blood, blood-forming organs and disorders involving the immune mechanism	1.2	1.4	1.2	1.1	1.1	1.2	1.1	1.7	0.86	1.55
• Diseases of the circulatory system	1.1	1.0	1.0	1.1	1.1	1.2	1.1	1.0	1.20	0.91
• Pregnancy, childbirth and the puerperium	0.5	0.4	0.5	0.5	0.6	0.8	0.3	1.8	2.00	6.00
**Diagnosis (ICD 10)**										
• Embedded and impacted teeth (K01)	9.9	11.2	10.6	9.9	9.5	8.4	10.8	5.2	0.75	0.48
• Chronic diseases of tonsils and adenoids (J35)	6.2	6.3	6.5	6.0	6.2	6.0	6.5	5.4	0.95	0.83
• Redundant prepuce, phimosis and paraphimosis (N47)	5.5	5.7	5.3	5.1	5.3	6.1	5.0	6.9	1.07	1.38
• Non suppurative otitis media (H65)	3.3	3.5	6.7	3.3	3.3	2.8	3.7	2.2	0.80	0.59
• Acute bronchiolitis (J21)	3.3	2.9	3.3	3.1	3.3	3.5	3.1	3.7	1.21	1.19
• Other gastroenteritis and colitis of infectious and unspecified origin (A09)	2.7	2.5	2.6	2.8	2.7	3.1	2.6	3.2	1.24	1.23
• Asthma (J45)	2.6	2.9	2.5	2.6	2.4	2.6	2.5	3.1	0.90	1.24
• Intracranial injury (S06)	2.1	1.9	2.1	2.1	2.2	1.9	2.1	1.9	1.00	0.90
• Viral and other specified intestinal infections (A08)	1.8	1.1	1.6	1.9	2.1	2.2	1.8	1.9	2.00	1.06
• Acute appendicitis (K35)	1.7	1.8	1.7	1.7	1.8	1.7	1.8	1.5	0.94	0.83
• Abdominal and pelvic pain (R10)	1.6	1.4	1.5	1.6	1.7	1.9	1.6	1.8	1.36	1.13
• Disorders related to short gestation and l ow birth weight, not elsewhere classified (P07)	1.6	1.6	1.6	1.6	1.6	1.7	1.6	1.8	1.06	1.13
• Other orthopaedic follow-up care (Z47)	1.5	1.5	1.5	1.5	1.5	1.5	1.6	1.4	1.00	0.88
• Dentofacial anomalies [including malocclusion] (K07)	1.5	1.4	1.5	1.5	1.6	1.2	1.6	0.8	0.86	0.50
• Fracture of forearm (S52)	1.4	1.4	1.5	1.5	1.4	1.4	1.5	1.1	1.00	0.73
• Acute tubulo-interstitial nephritis (N10)	1.4	1.3	1.3	1.4	1.4	1.4	1.4	1.3	1.08	0.93
• Fever of other and unknown origin (R50)	1.2	1.3	1.2	1.2	1.2	1.3	1.2	1.4	1.00	1.17
• Respiratory distress of newborn (P22)	1.1	1.2	1.1	1.1	1.0	1.0	1.1	1.0	0.83	0.91
• Dental caries (K02)	1.0	0.6	0.8	1.1	1.2	1.3	0.7	2.1	2.17	3.00
• Undescended testicle (Q53)	1.0	0.9	1.0	1.0	1.0	1.0	1.0	0.9	1.11	0.90

Note CMUc: complementary universal health insurance coverage

Concerning other frequent ICD-10 diagnoses (Table 3), 9.9% of admitted children had a diagnosis of embedded or impacted teeth but less for those who were more deprived (rQ5/Q1 = 0.75). The opposite was true for dental caries (10%) (rQ5/Q1 = 2.17). Children living in the most deprived quintile were more likely to be hospitalised for viral and other specified intestinal infections (rQ5/Q1 = 2.00), abdominal and pelvic pain (rQ5/Q1 = 1.36), and gastroenteritis, colitis, and infections of unspecified origin (rQ5/Q1 = 1.24). They were less likely to be hospitalized for non-suppurative otitis media (rQ5/Q1 = 0.80).

### Universal complementary health coverage (CMUc)

According to the level of socioeconomic precarity below the poverty threshold, represented by the CMUc status, the results were most often similar to those for the FDep (most deprived quintile Q5 with 30% of children with CMUc), apart from those linked to psychiatric pathologies and certain hospital diagnoses, suggesting a low-income effect. Indeed, certain LTD diagnoses showed a greater gap between those with CMUc and those without than by FDep quintile. This was true for mixed disorders of conduct and emotions (rCMUc/Not = 2.75), mixed specific developmental disorders (rCMUc/Not = 2.57), unspecified mental retardation (rCMUc/Not = 2.31), and pervasive developmental disorders (rCMUc/Not = 2.04) (Table 1).

For psychiatric hospitalisation, there was an increase in the frequency of admission according to the level of social deprivation (rCMUc/Not = 3.50, 0.7 vs 0.2%) (Table 2). Among them, the proportion of children with at least one admission increased rapidly between two and six years of age, reaching 0.76%, which was followed by a second increase between 11 and 16 years of age to close to 1.3%, which was more marked for children from families with CMUc (Fig 2). For hospitalisation diagnoses, this was also true for mental and behavioral disorders, (rCMUc/Not = 1.83, 4.4% vs 2.4%) (Table 3).

Other diseases coded as primary diagnoses for SSH stays showed a larger gap, such as pregnancy, childbirth, and puerperium diseases (rCMUc/Not = 6.00). Among girls without CMUc, more than 80% of hospitalisation for pregnancy, childbirth, and puerperium diseases were related to abortion. This proportion was approximately 30% for girls with CMUc.

## Discussion

This national observational study on the annual use of healthcare services, long-term chronic diseases (LTDs), and hospital diagnoses of children < 18 years of age in Metropolitan France in 2018 shows differences according to social deprivation, based on a geographic socio-demographic index, and precarity, with an annual income below the poverty line based on their having universal complementary medical insurance (CMUc). CMUc was allocated to 17.5% of the children in the overall population, with an increase across deprivation quintiles (Q5 (most deprived): 29.7%). Children in families with a high deprivation index value were more likely to have a LTD, such as a psychiatric disease. Relative to less deprived children, they were less likely to have seen a paediatrician, another specialist, or a dentist. Similarly, we observed a decreasing density of specialists and paediatricians, stable GP density, and increasing nurse density with increasing FDep. Children in families with a high deprivation index value also had more frequent ED visits, followed or not by a hospital admission, and mainly hospitalisations > 1 night.

### Chronic diseases and hospital diagnoses

Many studies on the prevalence of physical and mental diseases among children have generally been based on parent or adult reported data, including a broad definition of symptoms and common chronic diseases, but with a possible classical bias of over-reporting of their children’s symptoms or illnesses. A study among children 17 years of age or younger seen in primary care clinics between 2016 and 2018 reported the top six identified chronic conditions: obesity/being overweight (37%), eczema (16%), asthma (13%), food allergies (5%), attention deficit-hyperactivity disorder (4%), and hypertension (4%) [28]. Importantly, certain LTDs are not classified as a LTD in France. Definitions have been proposed to group children with medically complex or chronic conditions [29–31], with a prevalence estimated to be between 13 and 18% and who also show healthcare inequities [31, 32]. Several studies have provided data on specific chronic diseases or not are more affected by socioeconomic positions based on and other diseases [4].

For this study, the LTD prevalence of 4% (0–17 years) is low relative to the above mentioned studies. Apart from epidemiological, insurance, and healthcare system differences, LTDs with 100% coverage focus specifically on chronic diseases of higher severity that require costly, regular, and long-term care and are potentially life-threatening or lead to disabilities. Nevertheless, an underestimation may have occurred because some patients may have not yet been diagnosed or had low-intensity symptoms, resulting in little or no use of healthcare services, the diagnosis had not yet been confirmed, or the parents may have refused the LTD diseases status for their children.

LTDs associated with deprivation among youths and adolescents in this study included diabetes, asthma, and mental diseases, as reported in other studies [28–31]. Being overweight can be registered as an LTD only if there is a complication (diabetes). Furthermore, our results suggest a lower prevalence or the underdiagnosis of certain conditions among deprived children, such as scoliosis, diseases of the ear and mastoiditis, and neoplasms. There may be many explanations for this observation, such as, for example, lower incidence, less access to healthcare or fewer visits to specialists, for whom the density decreases across deprivation quintiles, and less treatment or higher mortality for tumours [32]. Our study also highlights a more marked association of mental disorders with deprivation based on the income level of the family (CMUc). A higher risk of having later secondary care-diagnosed mental disorders for children born to lower-income families vs those born to higher-income families has been reported [33].

Finally, we found a higher proportion of pregnancy, childbirth, and puerperium hospital diagnoses for the most deprived children (rQ5/Q1 = 2.00) mainly for those with CMUc (rCMUC/No = 6.00). This finding is consistent with those of other studies, as teenage pregnancies are known to be associated with a low socioeconomic status [34]. Moreover, a study on French pregnant women who gave birth showed that 75% of those < 18 years of age were beneficiaries of CMUc [23]. In addition, in our study, teenage women with CMUc had fewer hospital abortions then those without, and consequently more births. In France, the law allows any pregnant woman who considers herself to be in a situation of distress to ask a doctor to terminate her pregnancy, whether she is an adult or a minor. Only the woman concerned may to make the request. All women in France have the same rights of access to abortion. The costs of abortion are covered by social security. Nevertheless, numerous factors may vary according to the socio-economic level: socio-cultural and clinical factors, such as the diagnosis of pregnancy and its timing, access to medical abortion (not included in this study) or to various contraceptive methods, including the morning-after pill, recently made free of charge for all ages.

### Outpatient visits

In Italy, parents reported, in 2014–2015, that 68% of their children (5–18 years) had a GP or paediatric visit, 66% a specialist visit, and 60% a dentist visit in the preceding year [35]. The absence of children between 0 and 4 years of age from this study may explain the differences in the results from our study, such as fewer GP/paediatrician and specialist visits and more dentist visits. In our study, children showed the same frequency of visits to a GP or paediatrician (88%) across the deprivation index. Nevertheless, in non-deprived areas, they more often visited a paediatrician relative to more deprived areas, which may result from a higher density of paediatricians in non-deprived areas. Thus, in this study, which included youths, approximately 10% of children of all ages had no visits to a GP or paediatrician during the year, less for the youngest children and those with CMUc. This gap may concern free consultations carried out in the network of maternal and child protection centres (PMI: protection maternelle infantile) without exhaustive notified reimbursement and, consequently, identification. These centres are particularly oriented towards women and children in need of particular attention and in vulnerable populations in disadvantaged areas [36]. The difference in outpatient consultations between disadvantaged and non-disadvantaged youths may therefore be less than that identified in this study. An analysis of the Elfe cohort was specifically performed for a report on the health of infants born in 2011 [17, 18]. The study found at least one consultation in a PMI with a generalist during the first year of life for 9% of children born in 2011 and 8% for the five following years, which may reduce the gap observed, even if some could have visited a PMI and a GP in the same year. Moreover, they also found an increase in PMI visits during the first year of life with the deprivation quintile (Q1 = 10.5% and Q5 = 18.3%) and at four years (5% vs 11.5% respectively). Nevertheless, in our study, the gap was most pronounced for pre- and post-adolescence, suggesting more frequent absence of a yearly GP visit, even though examinations covered 100% by the national health insurance fund are proposed to parents for their youths and an examination every two years is planned between 7 and 16 years of age.

In France, the number of nurses on 1 January 2021 had doubled since 1999. Over the past several years, coercive measures have been taken to restrict the installation of nurses (2012), midwives (2012), and liberal masseur-physiotherapists (2012–2014, 2017) in the areas in which they are the most numerous, in addition to the traditional financial incentives in deficient territories (2005). For doctors, measures have included quantitative regulation of training, financial incentives, and measures to improve the conditions of practice [37].

The greatest decrease in specialist visits with deprivation was found for psychiatrists, whereas deprived children had more LTDs and SSH diagnoses for mental diseases and admissions to psychiatric hospitals, with two peaks. The first occurred at approximately 10 years of age, mainly for LTDs, and the second larger peak arose for children from 11 to 16 years of age with perhaps other acute or chronic psychiatric diseases, such as depression, pervasive developmental disorders, or eating disorders [38]. However, children can be exclusively followed by medical-psycho-pedagogical or medico-psychological centres with the visits supported by the centres and thus not identifiable. These are structures for children and adolescents with learning difficulties, or psychological, psychomotor, or behavioural disorders. Almost 90% of the children treated in these centres are between 5 and 14 years of age and most are boys [39]. In the last survey in 2003, the most frequent principal diagnosis was neurotic disorders (39%), most with a predominance of anxiety or inhibition in the field of learning, followed by developmental and instrumental disorders (18%) and progressive or behavioural disharmony (16%). Thus, there is an underestimation of the use of psychiatric consultations, which could be greater for disadvantaged children, who more frequently have a psychiatric LTD and are more likely to use this type of centre.

Children with a high level of deprivation were also less likely to have visited a dentist and up to three times more likely to have been hospitalized for dental caries but less likely for embedded and impacted teeth. Such inequality for access to dental healthcare has been previously reported [40]. In the Elfe study the prevalence of dental caries was associated with deprivation (FDep): at five years of age Q5 = 18.3%, Q1 = 6.3% (rQ5/Q1 = 2.9) and in current study the rQ5/Q1 = 0.86 for visits and 0.71 for dentist density [18]. In addition, the peaks at 6, 9, 12, and 15 years suggest the impact of the specific programme (M’T dents, 2007) of the French health insurance scheme.

In our study, we found that the density of healthcare professionals decreases with deprivation, except for GPs (stable), and nurses (increase). The result was the same for the frequency of annual visits for each type of healthcare professional, suggesting a relationship between visits once a year and density. This point is still a matter of debate, particularly in relation to factors that may or may not be associated with deprivation, such as age, neighbourhood, ethnicity, long-term conditions, mental health problems, insurance, etc. [5, 11, 12, 31, 39]. The ongoing debate notwithstanding, the reasons underlying the variance in the density of healthcare professionals between regions are the issue that needs to be understood.

### Emergency department visits and hospitalisation

We found that approximately 24% of children < 18 years of age had at least one ED visit, which is highly congruent with other estimations for 0- to 15-year-old children in France (more than 25%) [41] and in in Northwest London (24%) [42]. However, there are large differences between countries. The frequency was reported to be 13% in Italy (5–18 years) [35] and, in the US between 2010 and 2014, 12% of children (< 17 years) had visited an ED at least once during a given year (18% for < 3 years of age) with differences depending on insurance coverage [43]. A specific result of our study is that most deprived people more often live in a municipality with an ED, as they more often have CMUc (half of children), which may facilitate their use. It has been reported that these areas also include frequent users of EDs [44].

Another study found that the use of EDs varies from 18% to 27% between ethnic groups [6]. In England the most deprived group among 0- to 14-year-olds showed fewer GP and outpatient visits and a higher number of ED visits and admissions [11]. Nevertheless, several studies have shown an association between non-urgent ED visits and the sociodemographic characteristics of the child and family: age, income, social health insurance, support for access to primary care, and the reassurance and convenience with the use of this mode of care available during holidays and weekends, leading to long-term conditions more often being followed by hospital admission [45–49]. In England, increasing the GP density was found to reduce emergency admissions in deprived areas for areas in which GPs are concentrated in larger practices [47].

Approximately 60% of children had no visits to a healthcare professional one month before an ED visit and this value was approximately 40% among the youngest. Nevertheless, during the first year of life, 11 examinations are recommended, a number fairly close to the number of visits to a GP or paediatrician in this study (median: 11 and the first quartile: 8) and 98% of children had at least one consultation. For children between the ages of one and two years, two consultations are covered. Thus, the lack of a visit the month before could have occurred. The impact of primary care access on ED utilization has also been discussed but the discussion focused on other factors [42–46].

Between 2009 and 2012, the standardized hospital admission rate per 100 person-years in seven European countries for children from 0 to 19 years of age varied between 9.4 for Spain and 19.6 for Germany, with France in sixth position, with 13.5 [50]. In Italy, 5% of children between 5 and 18 years of age in 2014 (16% with chronic conditions) had a hospital admission, less than in our study which included children from 0 to 4 years of age (24% and 9%, respectively) [35].

The INSEE report for 2019, among children less than one year, a preliminary rate of 3.2 deaths p 1000 infants born alive for 2.1 in our study [26]. INSEE data were slightly higher for others age bands. However, for each class bands, an over mortality was found with deprivation and more important for infants even there is limitations. Over mortality, was frequently reported as for children according to neighbourhood income [8].

### Strengths and limitations

The main strength of this study was the use of the SNDS, which allowed us to include more than 13 million children, representing 94.4% of the metropolitan French population listed by the INSEE. Nevertheless, we observed a difference from the INSEE population that increases with age, which could be due to the non-inclusion of children who had no reimbursements during the year in our study and therefore led to a slight overestimation of healthcare consumption. Most appear to be adolescents with no reimbursements during the year. In addition, children born alive but not discharged from the hospital may have not been included in the population because they did not have an outpatient refund and, above all, their CMUc status could not be identified, but it may have been more frequent. For children < 6 years of age, we may have underestimated the proportion who visited a GP or paediatrician at least once due to the non-identification of consultations that occurred at a PMI. Nevertheless, many children may also visit at least one GP outside a PMI during the year. Other strengths of our study were the consideration of all major healthcare services together for age bands from 0 to 17 years and healthcare use spanning one year to avoid seasonal variation. The disadvantage indicator we used in this study is an indicator at the level of the smallest administrative unit. It does not necessarily prejudge the social disadvantage of each individual living in the municipality, and it was not totally independent of the CMUc at the individual level, which was also more frequent in more deprived quintiles. Moreover, among the oldest children, some may have been covered by CMUc before the study and exposed to deprivation factors, but coverage may have stopped before the study due to a positive evolution in annual family income.

## Conclusions

This study has relevant information on the characteristics, use of outpatient and inpatient healthcare services, and long-term chronic diseases of children according to deprivation levels in France. Deprived children more often had LTDs, including psychiatric diseases, and more often made use of EDs and the hospital and less use of dentists and specialists, such as psychiatrists, for whom the density decreases with deprivation. Thus, the utilisation of certain healthcare services appears to be suboptimal [51]. Preliminary results have been included in the annual social security report for use by MPs in voting on budgets. The main points were to strengthen the partnership between Health Insurance and PMIs to ensure the comprehensive monitoring of all children and strengthen government actions for those who are vulnerable, such as the deployment of new means to combat medical deserts and territorial inequalities in access to care, the proposal of a single simplified contract for installation in under-dense areas via regular "advanced consultations" outside their own practice areas, and the improvement of access to "unscheduled care" during the day to relieve the congestion of emergency rooms.

## Supporting information

S1 Table Time between the last outpatient visit with a general practitioner or paediatrician before an emergency department visit and the first one after an emergency department visit for those not hospitalised among children < 18 years of age in 2018 and followed for one year after their birth or birthday by social deprivation index and complementary universal health insurance coverage. *Same day: It is not possible to know whether the ED visit occurred before or after the GP or pediatrician visit. We considered that the GP or paediatrician visit occurred before the ED visit. CMUc: complementary universal health insurance coverage, SSH: short stay hospital. ED: emergency department.
(DOCX)
