# Supplementary material for: Social deprivation and the use of healthcare services over one year by children less than 18 years of age in 2018: A French nationwide observational study
Source: PLoS One. 2023 May 24;18(5):e0285467. doi: 10.1371/journal.pone.0285467 (PMC10208476; doi:10.1371/journal.pone.0285467)
Supplement: S1 Table — *Same day: It is not possible to know whether the ED visit occurred before or after the GP or pediatrician visit. We considered that the GP or paediatrician visit occurred before the ED visit. CMUc: complementary universal health insurance coverage, SSH: short stay hospital. ED: emergency department. (DOCX) [file pone.0285467.s001.docx]

Table S1. Supporting material

| Time between the last outpatient visit with a general practitioner or paediatrician before an emergency department visit and the first one after an emergency department visit for those not hospitalised among children < 18 years of age in 2018 and followed for one year after their birth or birthday by social deprivation index and complementary universal health insurance coverage.   \|  \|  \| **Social deprivation index (quintiles)**  (least deprived Q1. most deprived Q5) \| \| \| \| \| **CMUc** \| \| \| --- \| --- \| --- \| --- \| --- \| --- \| --- \| --- \| --- \| \|  \| **Total** \| **Q1** \| **Q2** \| **Q3** \| **Q4** \| **Q5** \| **Without** \| **With** \| \|  \| % \| % \| % \| % \| % \| % \| % \| % \| \| **All ED visit N (millions)** \| 4.805 \| 0.762 \| 0.872 \| 0.969 \| 0.986 \| 1.165 \| 3.577 \| 1.222 \| \| **Same day*** \| 8.8 \| 9.4 \| 10.0 \| 9.2 \| 8.5 \| 7.4 \| 9.1 \| 8.1 \| \| **Before** \|  \|  \|  \|  \|  \|  \|  \|  \| \| 1 day before \| 4.3 \| 4.7 \| 4.7 \| 4.4 \| 4.2 \| 4 \| 4.3 \| 4.5 \| \| 2-3 days \| 5.5 \| 5.8 \| 5.8 \| 5.5 \| 5.3 \| 5.4 \| 5.5 \| 5.7 \| \| 4-7 days \| 6.1 \| 6.1 \| 6.2 \| 6.1 \| 6.0 \| 6.1 \| 5.9 \| 6.7 \| \| 8-30 days \| 19.9 \| 19.9 \| 20 \| 19.9 \| 19.6 \| 20.2 \| 19.4 \| 21.6 \| \| No visit between 30 days and the day before \| 55.2 \| 54.0 \| 53.3 \| 54.9 \| 56.4 \| 56.8 \| 55.8 \| 53.4 \| \| **ED visit followed by SSH N (millions)** \| 0.527 \| 0.082 \| 0.095 \| 0.105 \| 0.110 \| 0.129 \| 0.386 \| 0.141 \| \| **Same day*** \| 14.7 \| 15.4 \| 16.2 \| 14.9 \| 14.7 \| 13.0 \| 15.7 \| 12.1 \| \| **Before** \|  \|  \|  \|  \|  \|  \|  \|  \| \| 1 day \| 7.4 \| 7.7 \| 7.8 \| 7.6 \| 7.3 \| 6.8 \| 7.7 \| 6.6 \| \| 2-3 days \| 8.0 \| 8.0 \| 8.3 \| 8.2 \| 7.8 \| 8.0 \| 8.3 \| 7.4 \| \| 4-7 days \| 7.1 \| 6.9 \| 7.1 \| 7 \| 6.9 \| 7.3 \| 7.0 \| 7.2 \| \| 8-30 days \| 19.2 \| 19.4 \| 19.2 \| 19.3 \| 18.9 \| 19.4 \| 18.9 \| 20.2 \| \| No visit between 30 days and the day before \| 43.5 \| 42.7 \| 41.4 \| 43.1 \| 44.3 \| 45.5 \| 42.4 \| 46.5 \| \| **ED visit not followed by SSH N (millions)** \| 4.278 \| 0.680 \| 0.776 \| 0.864 \| 0.877 \| 1.036 \| 3.191 \| 1.082 \| \| **Same day*** \| 8.1 \| 8.7 \| 9.3 \| 8.5 \| 7.7 \| 6.8 \| 8.3 \| 7.6 \| \| **Before** \|  \|  \|  \|  \|  \|  \|  \|  \| \| 1 day \| 4.0 \| 4.3 \| 4.3 \| 4 \| 3.8 \| 3.6 \| 3.9 \| 4.2 \| \| 2-3 days \| 5.2 \| 5.5 \| 5.5 \| 5.2 \| 5.0 \| 5.1 \| 5.2 \| 5.5 \| \| 4-7 days \| 6.0 \| 6.0 \| 6.1 \| 6.0 \| 5.8 \| 6.0 \| 5.8 \| 6.6 \| \| 8-30 days \| 20.0 \| 20.0 \| 20.1 \| 20.0 \| 19.7 \| 20.3 \| 19.5 \| 21.7 \| \| No visit between 30 days and the day before \| 56.7 \| 55.4 \| 54.8 \| 56.3 \| 57.9 \| 58.3 \| 57.4 \| 54.3 \| \| **After** \|  \|  \|  \|  \|  \|  \|  \|  \| \| 1 day \| 3.5 \| 3.3 \| 3.6 \| 3.4 \| 3.4 \| 3.5 \| 3.3 \| 3.9 \| \| 2-3 days \| 5.9 \| 6.0 \| 6.2 \| 5.9 \| 5.7 \| 5.9 \| 5.8 \| 6.4 \| \| 4-7 days \| 7.4 \| 7.5 \| 7.6 \| 7.4 \| 7.3 \| 7.4 \| 7.3 \| 8.0 \| \| 8-30 days \| 23.3 \| 23.8 \| 23.8 \| 23.4 \| 22.8 \| 22.9 \| 22.8 \| 24.7 \| \| No visit between the next day and 30 days after \| 59.9 \| 59.3 \| 58.8 \| 59.8 \| 60.8 \| 60.3 \| 60.8 \| 57.0 \|   *Same day: It is not possible to know whether the ED visit occurred before or after the GP or paediatrician visit. We considered that the GP or paediatrician visit occurred before the ED visit  CMUc: complementary universal health insurance coverage  SSH: short stay hospital  ED: emergency department |  |  |
| --- | --- | --- | --- | --- | --- | --- | --- | --- | --- | --- | --- | --- | --- | --- | --- | --- | --- | --- | --- | --- | --- | --- | --- | --- | --- | --- | --- | --- | --- | --- | --- | --- | --- | --- | --- | --- | --- | --- | --- | --- | --- | --- | --- | --- | --- | --- | --- | --- | --- | --- | --- | --- | --- | --- | --- | --- | --- | --- | --- | --- | --- | --- | --- | --- | --- | --- | --- | --- | --- | --- | --- | --- | --- | --- | --- | --- | --- | --- | --- | --- | --- | --- | --- | --- | --- | --- | --- | --- | --- | --- | --- | --- | --- | --- | --- | --- | --- | --- | --- | --- | --- | --- | --- | --- | --- | --- | --- | --- | --- | --- | --- | --- | --- | --- | --- | --- | --- | --- | --- | --- | --- | --- | --- | --- | --- | --- | --- | --- | --- | --- | --- | --- | --- | --- | --- | --- | --- | --- | --- | --- | --- | --- | --- | --- | --- | --- | --- | --- | --- | --- | --- | --- | --- | --- | --- | --- | --- | --- | --- | --- | --- | --- | --- | --- | --- | --- | --- | --- | --- | --- | --- | --- | --- | --- | --- | --- | --- | --- | --- | --- | --- | --- | --- | --- | --- | --- | --- | --- | --- | --- | --- | --- | --- | --- | --- | --- | --- | --- | --- | --- | --- | --- | --- | --- | --- | --- | --- | --- | --- | --- | --- | --- | --- | --- | --- | --- | --- | --- | --- | --- | --- | --- | --- | --- | --- | --- | --- | --- | --- | --- | --- | --- | --- | --- | --- | --- | --- | --- | --- | --- | --- | --- | --- | --- | --- | --- | --- | --- | --- | --- | --- | --- | --- | --- | --- | --- | --- | --- | --- | --- | --- | --- | --- | --- | --- | --- | --- | --- | --- | --- | --- | --- | --- | --- | --- | --- | --- | --- | --- | --- | --- | --- | --- | --- | --- | --- | --- | --- | --- | --- | --- | --- | --- | --- | --- | --- | --- | --- | --- |
